# Supplementary material for: Functions of Gle1 are governed by two distinct modes of self-association
Source: J Biol Chem. 2021 Jan 13;295(49):16813–25. doi: 10.1074/jbc.RA120.015715 (PMC7864074; doi:10.1074/jbc.RA120.015715)
Supplement: Supplementary file 1 [file mmc1.pdf]

Table S1. Plasmids

Plasmid	Description	Source
CP3994	Mammalian expression of *EGFP*	Aditi et al., 2015
pSW3909	Mammalian expression of *EGFP-Gle1A*	Aditi et al., 2015
pSW3908	Mammalian expression of *EGFP-Gle1B*	Folkman et al., 2013
pSW4537	Mammalian expression of *EGFP-gle1A- (8D)*	This Study
pSW4546	Mammalian expression of *EGFP-gle1A-Δ45-54*	This Study
pSW4553	Mammalian expression of *EGFP-gle1A-Δ45-54 (8D)*	This Study
pSW4535	Mammalian expression of *EGFP-gle1B- (8D)*	This Study
pSW4547	Mammalian expression of *EGFP-gle1B-Δ45-54*	This Study
pSW4552	Mammalian expression of *EGFP-gle1B-Δ45-54 (8D)*	This Study
pSW4563	Mammalian expression of *EGFP-gle1B-Δ45-54 ILLIYSASFLY*	This Study
CP3355	Mammalian expression of *mCherry*	Aditi et al., 2015
pSW4161	Mammalian expression of *mCherry-Gle1A*	Aditi et al., 2015
pSW4570	Mammalian expression of *mCherry-gle1A- (8D)*	This Study
pSW4571	Mammalian expression of *mCherry-gle1A-Δ45-54*	This Study
pSW4572	Mammalian expression of *mCherry-gle1A-Δ45-54 (8D)*	This Study
pSW4533	Bacterial expression of *SspB-gle1-152-360*	This Study
pSW4392	Bacterial expression of *His-MBP-(PPS)-gle1-1-360*	Aditi et al., 2018
pSW4509	Bacterial expression of *His-MBP-(PPS)-gle1-1-152*	This Study
pSW4556	Bacterial expression of *His-MBP-(PPS)-gle1-1-360 (8D)*	This Study
pSW4532	Bacterial expression of *His-MBP-(PPS)-gle1-1-360-Δ45-54*	This Study
pSW4555	Bacterial expression of *His-MBP-(PPS)-gle1-1-360-Δ45-54 (8D)*	This Study
pSW4554	Bacterial expression of *His-MBP-(PPS)-gle1-45-54 152-360*	This Study
pSW4494	Bacterial expression of *His-MBP-(PPS)-gle1-152-360*	This Study
pSW4529	Bacterial expression of *His-MBP-(PPS)-gle1-152-360 (8D)*	This Study
pSW422	Bacterial expression of *His-MBP-(PPS)-gle1-152-360 L248D L251D*	This Study
pSW4523	Bacterial expression of *His-MBP-(PPS)-gle1-152-360 L248D L251D L262D L315D M318D*	This Study
pSW4524	Bacterial expression of *His-MBP-(PPS)-gle1-152-360 L248D L251D L258D L262D L315D M318D*	This Study
CP3977	Bacterial expression of *His-MBP-(PPS)*	Center for Structural Biology, Vanderbilt University
	(8D): L248D L251D M255D L258D L315D M318D L322D L325D	

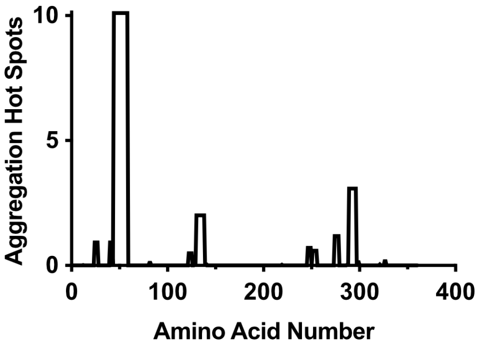

Figure S1. Characterization of an aggregation prone region swap of Gle1. Results of gle1B-Δ45-54 ILLIYSASFLY sequence submitted to AGGRESCAN server reveals a potential aggregation prone region at the generic aggregation sequence insertion.

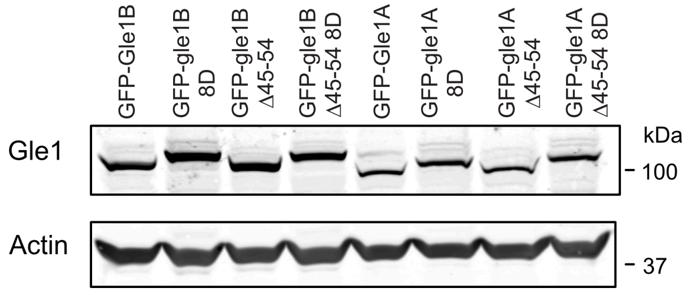

Figure S2. Cell lysates of HeLa cells expressing either *GFP-GLE1B, GFP-gle1B-8D, GFP-gle1B-Δ45-54, GFP-gle1B-Δ45-54-8D, GFP-GLE1A, GFP-gle1A-8D, GFP-gle1A-Δ45-54,* or *GFP-gle1A-Δ45-54-8D* were resolved on SDS-PAGE and immunoblotted with an anti-GFP.
